# Concurrent Alcohol Use and the Relative Risk of Community‐Acquired Pneumonia Associated With Anticholinergic and Non‐Anticholinergic Neurocognitively Active Medication Receipt: A National Nested Case–Control Study Among US Veterans

**DOI:** 10.1002/pds.70279

**Published:** 2025-12-03

**Authors:** William H. Wang, Kristina Crothers, Kathleen M. Akgün, Kirsha S. Gordon, Maria C. Rodriguez‐Barradas, Julie A. Womack, Jennifer Thompson, Amy C. Justice, Christopher T. Rentsch

**Affiliations:** ^1^ London School of Hygiene & Tropical Medicine London UK; ^2^ VA Puget Sound Health Care System and University of Washington Seattle Washington USA; ^3^ Yale School of Medicine New Haven Connecticut USA; ^4^ VA Connecticut Healthcare System West Haven Connecticut USA; ^5^ Michael E. DeBakey VA Medical Center and Baylor College of Medicine Houston Texas USA; ^6^ Yale School of Nursing West Haven Connecticut USA; ^7^ Yale School of Public Health New Haven Connecticut USA

**Keywords:** alcohol drinking, anticholinergic agents, case–control studies, drug interactions, pneumonia, psychotropic drugs, veterans

## Abstract

**Purpose:**

Anticholinergic medications and alcohol each independently increase the risk of community‐acquired pneumonia (CAP). Whether non‐anticholinergic neurocognitively active medications also increase risk, and if alcohol modifies these associations, remains unclear.

**Methods:**

We conducted a nested case–control study using Veterans Aging Cohort Study (VACS)‐National data. We identified 157 185 incident CAP cases requiring hospitalization between 2010 and 2022. Cases were matched 1:5 to controls without CAP on demographics, cohort entry date, and dwell time in the underlying cohort study using incidence density (risk‐set) sampling. CAP index date was hospital admission for cases and the equivalent follow‐up date for controls. Primary exposures were receipt of anticholinergic and non‐anticholinergic neurocognitively active medications within 90 days prior to the index date. Concurrent alcohol use was based on self‐reported measures in the year prior to the index date. We estimated odds ratios (ORs) for associations between medication use, alcohol consumption, and CAP using logistic regression, adjusting for confounders.

**Results:**

Median age was 69 years (interquartile range 62–78); 97% were male. Both medication types were independently associated with increased odds of CAP (anticholinergic: OR 1.62, 95% CI 1.57–1.67; non‐anticholinergic: OR 1.61, 95% CI 1.57–1.66). Concurrent alcohol use modified these associations. For anticholinergics, ORs were 1.74 (95% CI 1.66–1.83) for at‐risk consumption and 2.13 (95% CI 1.96–2.31) for hazardous/binge consumption. For non‐anticholinergics, ORs were 1.74 (95% CI 1.67–1.81) and 2.20 (95% CI 2.06–2.34), respectively.

**Conclusions:**

Non‐anticholinergic neurocognitively active medications showed similar CAP association patterns as anticholinergics, with the highest odds among those consuming alcohol. These findings highlight the need for caution when prescribing these medications and incorporating alcohol use into risk–benefit assessments.

## Summary

- Anticholinergic medications are known to increase the risk of community‐acquired pneumonia (CAP), but the role of non‐anticholinergic neurocognitively active medications is less well understood.
- Using electronic health record data on > 900 000 patients nationwide, we found similar patterns of associations with CAP for both anticholinergic and non‐anticholinergic neurocognitively active medications.
- Odds were further increased among those reporting concurrent alcohol consumption, with the strongest associations observed at hazardous or binge drinking levels.
- A dose–response relationship was observed with increasing numbers of concurrent neurocognitively active medications.
- Clinicians should incorporate alcohol use when weighing the risks and benefits of prescribing neurocognitively active medications.

## 1 Introduction

Anticholinergic agents include a range of medications [1, 2, 3] that block the neurotransmitter acetylcholine and have been prescribed to treat a variety of conditions [3, 4]. There is strong evidence of an association between the use of anticholinergics and an increased risk of community‐acquired pneumonia (CAP), resulting from anticholinergic effects on immunosuppression, sedation leading to aspiration and reduced or weakened cough response, and modified alveolar macrophage function [1, 3, 5, 6, 7, 8, 9, 10, 11]. A recent meta‐analysis found that anticholinergic use was associated with a 60% increased risk of CAP [12]. Due to safety concerns, the American Geriatric Society's Beers Criteria caution against the use of anticholinergics and advise the use of non‐anticholinergic alternatives for high‐risk subpopulations including older adults and those living with multiple chronic conditions [7].

Many classes of other neurocognitively active medications (e.g., antidepressants, antipsychotics) also have anticholinergic properties that can result in adverse effects such as dry mouth, thickened mucous secretion, cognitive impairment, and sedation [3, 5, 6]. While there is accumulating evidence for an association between neurocognitively active medications, such as antipsychotics and benzodiazepines, and increased risk of CAP in adults [13], evidence is limited and inconsistent for other classes of neurocognitively active medications [14]. Concurrent alcohol use may also exacerbate any potential effects. Alcohol is strongly associated with an increased risk of CAP both directly through impacts on lung inflammation and immune function, and indirectly through liver‐related complications associated with alcohol misuse such as alcoholic liver disease [15, 16, 17, 18, 19, 20]. Results from a 2018 meta‐analysis of 14 studies found that CAP risk was 82% higher in individuals who consumed higher levels of alcohol when compared to those who consumed lower levels of or no alcohol [9].

This study aimed to assess the role of concurrent alcohol use in the associations between anticholinergic and non‐anticholinergic neurocognitively active medication receipt and CAP. Although anticholinergic medications have well‐described associations with pneumonia, we included anticholinergic medications in the present study to enable direct comparison of their associations with community‐acquired pneumonia (CAP) within a single data source, study design, and analytic framework. We hypothesized that those with heavier alcohol consumption would have elevated odds of CAP compared to those with lower alcohol consumption for both medication groups.

## 2 Methods

### 2.1 Study Design and Data Source

**FIGURE 1:**
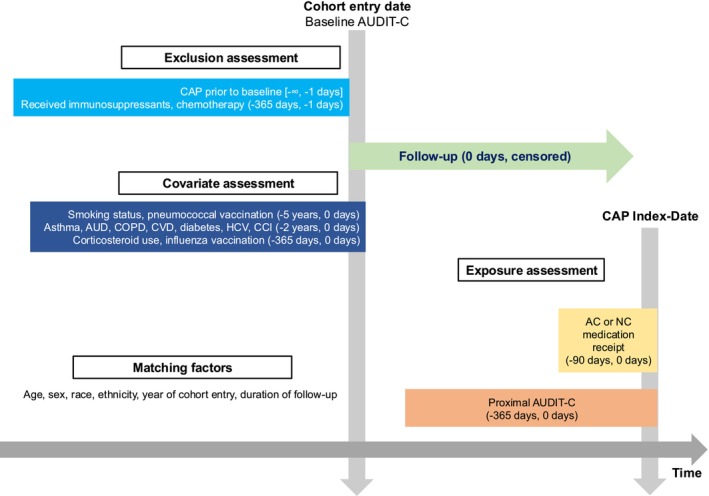
Study diagram. Patients were censored at the earliest of: incident CAP, date of death, one year after last VA visit, or end of study period. AC, anticholinergic; AUD, alcohol use disorder; AUDIT‐C, Alcohol Use Disorders identification Test‐Consumption; CAP, community‐acquired pneumonia; CCI, Charlson Comorbidity Index; COPD, chronic obstructive pulmonary disorder; CVD, cardiovascular disease; HCV, hepatitis C virus; NC, non‐anticholinergic neurocognitively active.

We conducted a nested case–control study in a US Veterans Administration cohort defined by members having completed at least one assessment of alcohol intake, a routine component of outpatient care. Specifically, we used data from the Veterans Aging Cohort Study‐National (VACS‐National), which includes > 13.5 million Veterans who ever received care in the US Department of Veterans Affairs (VA). The VA is the largest integrated healthcare system in the US serving > 9 million Veterans annually at > 1300 hospitals, medical centers, and community outpatient clinics nationwide [21]. All care is recorded in an electronic health record with daily uploads into the VA Corporate Data Warehouse. Available data include information on all outpatient and inpatient encounters, including demographics, diagnoses, pharmacy dispensing records, laboratory measures, procedures, vital signs, and routinely collected measurement of smoking and alcohol consumption. Figure 1 shows a graphical depiction of the study design in accordance with RECORD‐PE guidelines [22, 23].

This study was approved by the institutional review boards of Yale University (ref #1506016006) and VA Connecticut Healthcare System (ref #AJ0013). It has been granted a waiver of informed consent and is compliant with the Health Insurance Portability and Accountability Act. Ethics approval for analysis was also obtained from the LSHTM MSc Research Ethics Committee (Reference Number: 29055).

### 2.2 Selection of Cases and Controls

We first created a base cohort of patients who received VA care between January 1st, 2010 and December 31st, 2020. We defined the cohort entry date (“baseline”) as the date of first measurement of alcohol consumption occurring within 30 days of any outpatient prescription (to ensure receipt of routine care from the VA) that was at least 12 months after the first VA visit. We considered the 12 months prior to the baseline date as the baseline period to establish medical history. We excluded patients who, during the baseline period, had a prior CAP diagnosis (defined below) or were exposed to any chemotherapy or immunosuppressive medication (other than systemic or inhaled corticosteroids) as this may increase CAP risk. We excluded patients with no follow‐up after the baseline date. The cohort was followed until the earliest of: incident CAP, date of death, 1 year after the last VA visit, or the end of the study period.

Cases were identified as individuals diagnosed with CAP during the study period and through December 31st, 2022, to allow for at least 2 years of follow‐up for all patients identified in the base cohort. CAP cases were identified from hospital discharge diagnosis using validated ICD‐9 and ICD‐10 codes, as the primary diagnosis or secondary to human immunodeficiency virus (HIV), sepsis, and respiratory failure, similar to previous studies [8, 24, 25]. For each case, we matched up to 5 controls (i.e., patients without CAP) using incidence density (risk‐set) sampling at the time of the case event on age (±365 days), sex, race, ethnicity, cohort entry date (±365 days), and dwell time in the underlying cohort study. We matched on baseline date and duration of observation to avoid potential bias induced by temporal trends or time in study. The event index date was considered the date of hospital admission for incident CAP for cases and the date corresponding to the same duration of time since baseline for matched controls.

### 2.3 Alcohol and Medication Exposures

Alcohol consumption was assessed in the year prior to the event index date using the Alcohol Use Disorders Identification Test—Consumption (AUDIT‐C), a three‐item questionnaire that ascertains quantity and frequency of alcohol use to detect heavy drinking and potential alcohol use disorder (routinely administered per VA protocol at outpatient visits) [26, 27]. Since 2007, the VA has required annual AUDIT‐C screening on all patients during routine healthcare visits in primary care, with screening as part of standard clinical practice (rather than triggered by clinical concern) [28]. AUDIT‐C scores range from 0 to 12 with the likelihood of physiologic injury and mortality increasing with higher AUDIT‐C score [29]. We categorized AUDIT‐C scores as: 0 for no current alcohol use, 1 to 3 for low, 4 to 7 for at‐risk, and ≥ 8 for hazardous/binge consumption [30]. Low consumption (AUDIT‐C = 1 to 3) was a priori selected as the referent group in all analyses, as previous work has shown that individuals reporting no current alcohol use are a heterogeneous group comprising very few lifetime abstainers, with most having quit drinking after alcohol‐related or other health problems [31]. This approach aligns with previous VA research [32, 33, 34] and is less prone to bias than comparing all other groups to this category, who are often at higher risk of comorbidity and mortality than those who report low consumption.

Exposure to anticholinergic and neurocognitively active medications was ascertained 90 days prior to the event index date, in line with previous work [35]. Due to the absence of a single system to classify and rank anticholinergic properties of different medicines, Salahudeen et al. conducted a systematic review to compile seven different scales into a single composite scale [36]. For the present study, we considered all 67 medications listed as having high anticholinergic burden by Salahudeen et al., with 57 of those included in the VA formulary. To ascertain exposure to other neurocognitively active medications, we extracted pharmacy records for all opioids, antipsychotics, lithium, anticonvulsants, anti‐Parkinson's, antidepressants, sedatives/hypnotics, muscle relaxants, amphetamine derivatives, antihistamines, and antivertigo agents.

A list of all medications included in this study can be found in Table S1. Neurocognitively active medications that were classified by Salahudeen et al. [36] as having high anticholinergic burden were categorized as an anticholinergic medication in this study. For example, scopolamine has anticholinergic properties and is the only neurocognitively active medication in the antivertigo subclass. For this paper, scopolamine was classified as anticholinergic. Therefore, our analysis compares anticholinergic with non‐anticholinergic neurocognitively active medications.

### 2.4 Covariates

We selected covariates that may confound the association between medications of interest, alcohol consumption, and CAP. Sociodemographic characteristics included age, sex, race, and ethnicity. Smoking status was categorized as current, former, or never using a validated algorithm [37]. Clinical characteristics, determined by the presence of ICD‐9 and ICD‐10 diagnostic codes in the baseline period, included alcohol use disorder (AUD), asthma, cardiovascular disease, chronic obstructive pulmonary disease (COPD), diabetes mellitus, and chronic hepatitis C virus infection. We additionally adjusted for overall comorbidity burden using the Charlson Comorbidity Index (CCI), which was assessed during the baseline period. The CCI includes diagnostic codes across 17 clinical domains, including acute myocardial infarction, congestive heart failure, peripheral vascular disease, cerebral vascular accident, dementia, pulmonary disease, connective tissue disorder, peptic ulcer, mild and severe liver disease, diabetes and diabetic complications, paraplegia, renal disease, cancer and metastatic cancer, and HIV/AIDS [38]. Receipt of inhaled or oral corticosteroids and influenza vaccination were assessed in the baseline period, and pneumococcal vaccination was assessed in the 5 years prior to baseline. We calculated the total number of outpatient medications (excluding primary exposures) dispensed to a patient 90 days prior to the event index date to account for potential confounding by polypharmacy.

### 2.5 Statistical Analysis

Descriptive statistics were calculated to characterize exposures, matching factors, and covariates between CAP cases and their matched controls. The incidence of CAP was estimated in the base cohort. We fit two conditional logistic regression models with CAP as the outcome to estimate odds ratios (ORs) and 95% confidence intervals (CIs) that accounted for the matched design. One model estimated the independent and interactive associations between anticholinergic medication receipt and alcohol consumption, and the second model did so for non‐anticholinergic neurocognitively active medication receipt and alcohol consumption. Each model included indicators for medication receipt (binary yes/no) and alcohol consumption (five categories: abstinent, low‐risk, at‐risk, hazardous/binge, and missing), as well as their interaction term. Minimally adjusted models included only matching factors (age, sex, race, ethnicity, year of cohort entry, duration of follow‐up), and fully adjusted models additionally adjusted for smoking status, alcohol use disorder, asthma, cardiovascular disease, chronic obstructive pulmonary disease, diabetes, chronic hepatitis C virus infection, Charlson Comorbidity Index, corticosteroid receipt, influenza and pneumococcal vaccination, and number of other chronic medications. Coefficients and standard errors from the fully parameterized models are provided in Tables S2 and S3. All analyses were conducted using the Stata MP v18.0 statistical software.

### 2.6 Secondary Analyses

First, we assessed for a dose–response association by increasing counts of medication use (i.e., 0, 1, 2, ≥ 3 for anticholinergic medication receipt; 0, 1, 2, 3, ≥ 4 for non‐anticholinergic neurocognitively active medication receipt) in the 90 days prior to event index date. These models replaced the binary indicator of any medication receipt with the corresponding count variable while retaining all other model specifications. Second, we analyzed each of the 11 classes of non‐anticholinergic neurocognitively active medications (listed above) in separate models to further investigate the biggest contributors to any observed association in the overall analysis. In these models, the binary indicator of any non‐anticholinergic neurocognitively active medication receipt from the primary analysis was replaced with a binary indicator for receipt (yes/no) of each specific medication class, retaining all other model specifications listed above.

### 2.7 Sensitivity Analyses

In the primary analysis, we included a missing category for 157 893 (17%) patients with missing AUDIT‐C data in the year prior to the event index date. A sensitivity analysis substituted missing proximal AUDIT‐C with information from the AUDIT‐C measurement that established entry into the underlying cohort. We performed this under the assumption that alcohol consumption patterns do not change dramatically in middle‐aged patients with a median duration of follow‐up < 3 years.

## 3 Results

### 3.1 Cohort Description

**FIGURE 2:**
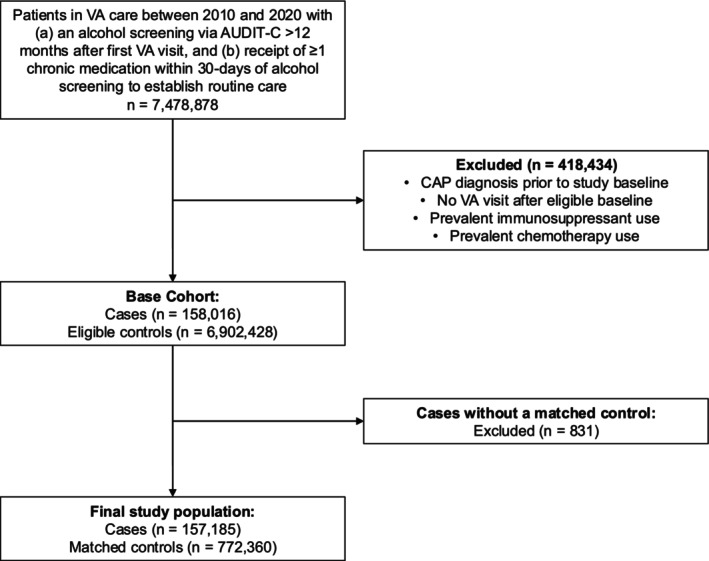
Study flowchart. AUDIT‐C, Alcohol Use Disorders Identification Test‐Consumption; CAP, community‐acquired pneumonia; VA, US Department of Veterans Affairs.

There were 7 478 878 patients who received VA care during the study period with an eligible AUDIT‐C measurement that established cohort entry (Figure 2). We excluded a total of 418 434 patients who had a previous CAP diagnosis, were exposed to any chemotherapy or immunosuppressive medication, and those who did not have a follow‐up visit. After applying exclusion criteria, 7 060 444 patients were eligible for matching, including 158 016 CAP cases. Only 831 CAP cases were not matched to at least one control. The final study population of 929 545 patients included 157 185 cases and 772 360 controls. Median age was 69 years (interquartile range [IQR] 62–78), 97% were male, 71% were White, 17% were Black, 7% were Hispanic, and median follow‐up was 2.3 years (IQR 0.9–4.4).

In the 90 days prior to CAP index date, 58,500 (37%) of cases and 137 651 (18%) of controls received an anticholinergic medication (Table 1). In the same period, 87 768 (56%) of cases and 271 543 (35%) of controls received a non‐anticholinergic neurocognitively active medication. More cases than controls reported no alcohol consumption in the year prior to event index date (64% of cases vs. 45% of controls). Fewer cases than controls reported low consumption (19% of cases vs. 27% of controls) and at‐risk consumption (6% of cases vs. 8% of controls), with similar prevalence of hazardous/binge consumption (2% of cases vs. 1% of controls).

**TABLE 1:** Characteristics of patients with incident community‐acquired pneumonia and their matched controls.

	Cases, no (%)	Controls, no (%)
	*n* = 157 185	*n* = 772 360
Matching factors		
Age at baseline, years (median [IQR])	69 (62–78)	69 (62–78)
Age at baseline, years		
20–60	27 540 (18%)	136 691 (18%)
61–70	55 541 (35%)	275 873 (36%)
71–80	38 582 (25%)	190 474 (25%)
≥ 81	35 522 (23%)	169 322 (22%)
Sex		
Male	152 871 (97%)	746 044 (97%)
Female	5314 (3%)	26 316 (3%)
Race and ethnicity		
White	110 717 (71%)	546 759 (71%)
Black/African American	27 391 (17%)	133 630 (17%)
Hispanic	10 918 (7%)	51 693 (7%)
Asian	576 (0.4%)	2808 (0.4%)
American Indian/Alaska Native	822 (0.5%)	4029 (0.5%)
Native Hawaiian/Pacific Islander	822 (0.5%)	4047 (0.5%)
Mixed race	1072 (0.7%)	5258 (0.7%)
Missing	4867 (3%)	24 136 (3%)
Year of baseline		
2010 to 2012	65 006 (41%)	315 102 (41%)
2013 to 2016	55 583 (35%)	274 339 (36%)
2017 to 2020	36 596 (23%)	182 919 (24%)
Duration of follow‐up, years (median [IQR])	2.3 (0.9–4.5)	2.2 (0.9–4.4)
Primary exposures		
Anticholinergic medication receipt	58 500 (37%)	137 651 (18%)
Non‐anticholinergic neurocognitively active medication receipt	87 768 (56%)	271 543 (35%)
Alcohol consumption measured by AUDIT‐C		
Abstinent (0)	99 996 (64%)	347 823 (45%)
Low‐risk (1–3)	30 114 (19%)	205 083 (27%)
At‐risk (4–7)	9896 (6%)	63 758 (8%)
Hazardous/binge (8–12)	3899 (2%)	11 083 (1%)
Missing	13 280 (8%)	144 613 (19%)
Clinical characteristics		
Smoking status		
Never	34 666 (22%)	248 461 (32%)
Former	53 286 (34%)	308 172 (40%)
Current	69 233 (44%)	215 727 (28%)
Alcohol use disorder	17 573 (11%)	41 636 (5%)
Asthma	7571 (5%)	19 587 (3%)
Cardiovascular disease	53 821 (34%)	115 634 (15%)
Chronic obstructive pulmonary disease	52 886 (34%)	74 143 (10%)
Diabetes	62 931 (40%)	240 152 (31%)
Chronic hepatitis C virus infection	8785 (6%)	19 710 (3%)
Charlson Comorbidity Index score		
0	24 609 (16%)	304 928 (39%)
1–2	59 840 (38%)	314 882 (41%)
3–4	39 378 (25%)	108 841 (14%)
≥ 5	33 358 (21%)	43 709 (6%)
Corticosteroid receipt in previous year	63 264 (40%)	159 397 (21%)
Influenza vaccination receipt in previous year	108 503 (69%)	486 788 (63%)
Pneumococcal vaccination receipt in previous 5 years	26 792 (17%)	118 243 (15%)
Number of other chronic medications^a^		
0	33 731 (21%)	217 043 (28%)
1–5	27 897 (18%)	283 996 (37%)
6–10	39 345 (25%)	176 703 (23%)
≥ 11	56 212 (36%)	94 618 (12%)

Abbreviations: AUDIT‐C, alcohol use disorder identification test‐consumption; IQR, interquartile range.

^a^ Excluding anticholinergic and non‐anticholinergic neurocognitively active medications.

### 3.2 Primary Analyses

Incidence of CAP in the base cohort was 4.6 events per 1000 person‐years. Anticholinergic medication receipt in the 90 days prior to event index date was strongly associated with CAP in the minimally adjusted model (OR 3.13, 95% CI 3.04–3.21), which attenuated after full adjustment (OR 1.62, 95% CI 1.57–1.67; Table 2). Similar patterns were observed for non‐anticholinergic neurocognitively active medication receipt (OR 2.74, 95% CI 2.68–2.81 in the minimally adjusted model; OR 1.61 95% CI 1.57–1.66 in the fully adjusted model).

**TABLE 2:** Independent associations and interactions between anticholinergic and non‐anticholinergic neurocognitively active medication receipt and alcohol consumption.

(A) Anticholinergic model	Minimally adjusted OR (95% CI)	Fully adjusted OR (95% CI)
Independent Associations		
Anticholinergic medication receipt		
No	1.00 (ref)	1.00 (ref)
Yes	3.13 (3.04–3.21)	1.62 (1.57–1.67)
Alcohol consumption		
Abstinent	1.97 (1.94–2.00)	1.71 (1.67–1.74)
Low‐risk	1.00 (ref)	1.00 (ref)
At‐risk	1.07 (1.04–1.11)	1.03 (1.00–1.06)
Hazardous/binge	2.54 (2.42–2.66)	1.77 (1.68–1.86)
Missing	0.60 (0.59–0.62)	0.61 (0.59–0.62)
Interactions		
Abstinent		
Medication receipt	4.87 (4.78–4.97)	2.24 (2.19–2.29)
Low‐risk consumption		
No medication receipt	1.00 (ref)	1.00 (ref)
Medication receipt	3.13 (3.04–3.21)	1.62 (1.57–1.67)
At‐risk consumption		
Medication receipt	3.51 (3.36–3.67)	1.74 (1.66–1.83)
Hazardous/binge consumption		
Medication receipt	5.94 (5.53–6.37)	2.13 (1.96–2.31)
Missing consumption		
Medication receipt	2.00 (1.92–2.08)	1.08 (1.03–1.13)

(B) Non‐anticholinergic neurocognitively active model	Minimally adjusted OR (95% CI)	Fully adjusted OR (95% CI)
Independent Associations		
Non‐anticholinergic neurocognitively active medication receipt		
No	1.00 (ref)	1.00 (ref)
Yes	2.74 (2.68–2.81)	1.61 (1.57–1.66)
Alcohol consumption		
Abstinent	2.00 (1.96–2.04)	1.76 (1.72–1.79)
Low‐risk	1.00 (ref)	1.00 (ref)
At‐risk	1.05 (1.02–1.09)	1.02 (0.98–1.06)
Hazardous/binge	2.83 (2.67–3.00)	1.98 (1.86–2.11)
Missing	0.63 (0.61–0.64)	0.60 (0.58–0.62)
Interactions		
Abstinent		
Medication receipt	4.65 (4.55–4.75)	2.35 (2.29–2.40)
Low‐risk consumption		
No medication receipt	1.00 (ref)	1.00 (ref)
Medication receipt	2.74 (2.68–2.81)	1.61 (1.57–1.66)
At‐risk consumption		
Medication receipt	3.11 (3.00–3.22)	1.74 (1.67–1.81)
Hazardous/binge consumption		
Medication receipt	5.48 (5.18–5.78)	2.20 (2.06–2.34)
Missing consumption		
Medication receipt	1.84 (1.77–1.90)	1.10 (1.06–1.14)

*Note:* p‐interaction between medication receipt and alcohol consumption for both models was < 0.0001. Two models were fit, one for anticholinergic (model A) and one for non‐anticholinergic neurocognitively active (model B) medication receipt, both displayed above. Each model included indicators for medication receipt (binary yes/no) and alcohol consumption (five categories: abstinent, low‐risk, at‐risk, hazardous/binge, and missing), as well as their interaction term. Minimally adjusted models included only matching factors (age, sex, race, ethnicity, year of cohort entry, duration of follow‐up). Fully adjusted models additionally adjusted for smoking status, alcohol use disorder, asthma, cardiovascular disease, chronic obstructive pulmonary disease, diabetes, chronic hepatitis C virus infection, Charlson Comorbidity Index, corticosteroid receipt, influenza and pneumococcal vaccination, and number of other chronic medications. Coefficients and standard errors from the fully parameterized models are provided in Tables S2 and S3.

Among patients who reported low‐risk alcohol consumption, anticholinergic medication receipt compared to no anticholinergic medication receipt was associated with 62% (OR 1.62, 95% CI 1.57–1.67) increased odds for CAP in the fully adjusted model (Table 2). The association between anticholinergic medication receipt and CAP was stronger for patients who reported concurrent at‐risk consumption (OR 1.74, 95% CI 1.66–1.83) and hazardous/binge consumption (OR 2.13, 95% CI 1.96–2.31). Similar findings were observed for neurocognitively active medication receipt (OR 1.61, 95% CI 1.57–1.66 among patients who reported low‐risk consumption; OR 1.74, 95% CI 1.67–1.81 among patients who reported at‐risk consumption; OR 2.20, 95% CI 2.06–2.34 among patients who reported hazardous/binge consumption), compared to patients who reported low‐risk alcohol consumption without neurocognitively active medication receipt.

### 3.3 Secondary Analyses

Compared to patients with no anticholinergic medication receipt, there was a dose–response with each additional anticholinergic medication received in the 90 days prior to event index date (OR 1.36, 95% CI 1.34–1.38 for one; OR 1.51, 95% CI 1.47–1.54 for two; and OR 1.77, 95% CI 1.70–1.84 for three or more; Table 3). Results were similar for the count of non‐anticholinergic neurocognitively active medications received (OR 1.29, 95% CI 1.26–1.31 for one; OR 1.42, 95% CI 1.39–1.45 for two; OR 1.59, 95% CI 1.55–1.63 for three; and OR 1.89, 95% CI 1.85–1.94 for four or more). The strongest neurocognitively active class‐specific associations with CAP were observed for opioids (OR 1.62, 95% CI 1.59–1.65) and antipsychotics (OR 1.55, 95% CI 1.49–1.61), with associations stronger than those observed with any neurocognitively active medication receipt in the primary analysis (Table 3). We also found evidence of independent associations between CAP and lithium (OR 1.31, 95% CI 1.18–1.46), anticonvulsants (OR 1.26, 95% CI 1.24–1.29), anti‐Parkinson's agents (OR 1.24, 95% CI 1.18–1.30), antidepressants (OR 1.20, 95% CI 1.18–1.22), sedatives/hypnotics (OR 1.17, 95% CI 1.15–1.20), muscle relaxants (OR 1.13, 95% CI 1.11–1.16), and other neurocognitively active medications (OR 2.77, 95% CI 2.65–2.90). We did not find evidence of independent associations between CAP and amphetamine derivatives (OR 1.11, 95% CI 0.99–1.24), and antihistamines (OR 0.99, 95% CI 0.96–1.01).

**TABLE 3:** Secondary analyses investigating the count and class of medications received in the 90 days prior to the event index date.

	Cases, no (%)	Controls, no (%)	Minimally adjusted OR (95% CI)	Fully adjusted OR (95% CI)
By count of medications received				
Count of anticholinergic medications received				
0	98 685 (63%)	634 709 (82%)	1.00 (ref)	1.00 (ref)
1	36 764 (23%)	99 749 (13%)	2.35 (2.32–2.39)	1.36 (1.34–1.38)
2	14 705 (9%)	28 532 (4%)	3.25 (3.18–3.32)	1.51 (1.47–1.54)
≥ 3	7031 (4%)	9370 (1%)	4.74 (4.59–4.90)	1.77 (1.70–1.84)
Count of non‐anticholinergic neurocognitively active medications received				
0	69 417 (44%)	500 817 (65%)	1.00 (ref)	1.00 (ref)
1	26 651 (17%)	116 568 (15%)	1.79 (1.76–1.82)	1.29 (1.26–1.31)
2	20 565 (13%)	68 576 (9%)	2.36 (2.32–2.40)	1.42 (1.39–1.45)
3	14 545 (9%)	38 373 (5%)	3.02 (2.96–3.09)	1.59 (1.55–1.63)
≥ 4	26 007 (17%)	48 026 (6%)	4.41 (4.33–4.50)	1.89 (1.85–1.94)
By class of non‐anticholinergic neurocognitively active medication received				
Opioids				
No	116 736 (74%)	685 180 (89%)	1.00 (ref)	1.00 (ref)
Yes	40 449 (26%)	87 180 (11%)	2.79 (2.75–2.83)	1.62 (1.59–1.65)
Antipsychotics				
No	151 541 (96%)	760 231 (98%)	1.00 (ref)	1.00 (ref)
Yes	5644 (4%)	12 129 (2%)	2.08 (2.01–2.15)	1.55 (1.49–1.61)
Lithium				
No	156 588 (99.6%)	7 706 870 (99.8%)	1.00 (ref)	1.00 (ref)
Yes	597 (0.4%)	1680 (0.2%)	1.56 (1.42–1.72)	1.31 (1.18–1.46)
Anticonvulsants				
No	121 156 (77%)	681 470 (88%)	1.00 (ref)	1.00 (ref)
Yes	36 029 (23%)	90 890 (12%)	2.12 (2.09–2.15)	1.26 (1.24–1.29)
Anti‐Parkinson's agents				
No	154 210 (98%)	764 159 (99%)	1.00 (ref)	1.00 (ref)
Yes	2975 (2%)	9201 (1%)	1.69 (1.62–1.77)	1.24 (1.18–1.30)
Antidepressants				
No	113 110 (72%)	644 721 (83%)	1.00 (ref)	1.00 (ref)
Yes	44 075 (28%)	127 639 (17%)	1.90 (1.87–1.92)	1.20 (1.18–1.22)
Sedative/hypnotics				
No	139 408 (89%)	721 798 (93%)	1.00 (ref)	1.00 (ref)
Yes	17 777 (11%)	50 562 (7%)	1.72 (1.68–1.75)	1.17 (1.15–1.20)
Muscle relaxants				
No	144 731 (92%)	738 042 (96%)	1.00 (ref)	1.00 (ref)
Yes	12 454 (8%)	34 318 (4%)	1.77 (1.73–1.81)	1.13 (1.11–1.16)
Amphetamine derivatives				
No	156 677 (99.7%)	770 419 (99.7%)	1.00 (ref)	1.00 (ref)
Yes	508 (0.3%)	1942 (0.3%)	1.22 (1.11–1.35)	1.11 (0.99–1.24)
Antihistamines				
No	137 950 (88%)	717 007 (93%)	1.00 (ref)	1.00 (ref)
Yes	19 235 (12%)	55 353 (7%)	1.71 (1.68–1.74)	0.99 (0.96–1.01)
Other				
No	151 930 (97%)	767 325 (99%)	1.00 (ref)	1.00 (ref)
Yes	5265 (3%)	5035 (1%)	4.93 (4.74–5.14)	2.77 (2.65–2.90)

*Note:* Each secondary analysis shown above used a separate model switching out the main exposure variable. For analyses by count of medications received, the binary indicator of any medication receipt from the primary models was replaced with a count variable representing the number of medications received in the 90 days prior to the event index date. For class‐specific analyses, the binary indicator of any non‐anticholinergic neurocognitively active medication receipt was replaced with a binary indicator (yes/no) for each of the 11 medication classes. P‐trend for both anticholinergic and non‐anticholinergic neurocognitively active medication counts was < 0.0001. Minimally adjusted models included only matching factors (age, sex, race, ethnicity, year of cohort entry, duration of follow‐up). Fully adjusted models additionally adjusted for smoking status, alcohol use disorder, asthma, cardiovascular disease, chronic obstructive pulmonary disease, diabetes, chronic hepatitis C virus infection, Charlson Comorbidity Index, corticosteroid receipt, influenza and pneumococcal vaccination, and number of other chronic medications. The “Other” category included the following 10 agents: belladonna, hyoscyamine, dicyclomine, propantheline, benztropine, trihexyphenidyl, procyclidine, atomoxetine, dextromethorphan, and metoclopramide.

### 3.4 Sensitivity Analyses

Among patients without missing proximal AUDIT‐C, there was 79% agreement between baseline and proximal AUDIT‐C. Sensitivity analyses substituting missing proximal AUDIT‐C with baseline AUDIT‐C provided consistent results to primary analyses for any anticholinergic medication receipt and low‐risk consumption (OR 1.69, 95% CI 1.64–1.74), at‐risk consumption (OR 1.78, 95% CI 1.69–1.86), and hazardous/binge consumption (OR 2.12, 95% CI 1.96–2.30). Similar consistency was observed for any non‐anticholinergic neurocognitively active medication receipt and low‐risk consumption (OR 1.71, 95% CI 1.67–1.76), at‐risk consumption (OR 1.82, 95% CI 1.76–1.90), and hazardous/binge consumption (OR 2.26, 95% CI 2.13–2.40).

## 4 Discussion

In this nationwide observational study, we found robust evidence of similar patterns of association with incident CAP for both anticholinergic and non‐anticholinergic neurocognitively active medications. We additionally found that these associations with both anticholinergic and non‐anticholinergic neurocognitively active medications were further modified by concurrent alcohol consumption, with patients who reported at‐risk and hazardous/binge consumption having greater odds of CAP. These results were further substantiated in sensitivity analysis that found odds for CAP increased in a dose–response fashion with each additional anticholinergic and non‐anticholinergic neurocognitively active medication received.

### 4.1 Findings in Context

Our finding of an association between anticholinergic medication and CAP is consistent with previous meta‐analyses [12]. While evidence for this association between anticholinergic medication and CAP is well established, that of non‐anticholinergic neurocognitively active medications and CAP is more limited. To date, existing studies have been limited to specific classes of neurocognitively active medications, including antipsychotics, benzodiazepines, and antidepressants. One study found that antipsychotic and benzodiazepine use but not antidepressant use was associated with increased risk of CAP, though limited power resulted in a high degree of uncertainty in these association [13]. Our work extended investigations to 10 classes of neurocognitively active medications in a large‐scale, national cohort that improved statistical power. Our study confirmed previous work finding independent associations between antipsychotics and sedative/hypnotics, and extended evidence for opioids, lithium, anticonvulsants, anti‐Parkinson's agents, and muscle relaxants. Where previous evidence was inconsistent with limited power, our study found strong evidence of an independent association between antidepressants and CAP. The elevated odds observed for the “Other” neurocognitively active medications category likely reflect reverse causation and residual confounding rather than a causal effect. Most exposures (96%) in this category were to dextromethorphan, a cough suppressant often used by patients with respiratory symptoms, suggesting that medication use may have occurred as a response to, rather than a cause of, early pneumonia symptoms. The remaining agents in this group, including belladonna alkaloids, dopaminergic agents, and other centrally acting drugs, are typically prescribed to patients with complex neurological, psychiatric, or gastrointestinal conditions, who may have a higher underlying risk of CAP. While our models adjusted for many key confounders, it is possible that not all relevant factors were fully captured for these agents.

Our study further identified interactions between concurrent alcohol use and receipt of both anticholinergic and non‐anticholinergic neurocognitively active medications. Results from a 2018 systematic review and meta‐analysis demonstrated that the risk of CAP was > 80% higher in individuals who consumed any or high levels of alcohol when compared to those who consumed lower or no alcohol [9]. Alcohol is thought to increase the risk of CAP by reducing oropharyngeal tone leading to increased aspiration, a known mechanism of pneumonia risk [1, 3, 9]. Alcohol consumption has also been shown to modify alveolar macrophage function, reducing pulmonary defenses against infection [9]. Our study found evidence that the odds of CAP associated with anticholinergic and non‐anticholinergic neurocognitively active medication receipt were further pronounced in patients who reported concurrent alcohol consumption, particularly at‐risk and hazardous/binge levels. These findings suggest that the mechanisms of CAP risk between alcohol and these medications may be synergistic. Our results highlight the need for further investigation and implementation of pneumonia prevention measures in this high‐risk population; these include well‐recognized strategies such as vaccination and smoking cessation efforts, but also monitoring of and intervention on alcohol use, a less recognized risk factor for CAP among patients prescribed neurocognitively active medications. Future research should aim to quantify medication interactions with other behavioral factors, such as smoking; doing so will require routinely collected, longitudinal data on smoking quantity and frequency at scale, which are not currently available in VA records.

### 4.2 Strengths and Limitations

This study's strengths included the availability and use of highly detailed, longitudinal, electronic health record data, including routinely collected measurements of alcohol consumption, available from the largest integrated health system in the US. The systematic and universal screening of alcohol consumption (rather than screening triggered by clinical concern) makes the VA one of the few healthcare systems of its size with data suitable for investigating medication‐alcohol interactions. Rigorous pharmacoepidemiology methods and findings robust to sensitivity analysis also added to the strengths of this observational study. We also recognize possible limitations. First, due to the observational nature of this study, a degree of uncertainty exists that can only be addressed with randomization. Nevertheless, we took steps to mitigate the potential for sources of bias and confounding through rigorous study design and adjustment for a large number of potential confounders. Second, the use of self‐reported alcohol consumption may have introduced information bias due to risks of social desirability and recall errors. Thus, some degree of underreporting or misclassification is likely. If such misclassification were non‐differential with respect to CAP risk, estimates would likely be biased toward the null; however, differential underreporting among individuals with characteristics associated with CAP risk could also lead to unpredictable bias in either direction. Third, the attenuation observed after full adjustment likely reflects confounding by comorbidity, polypharmacy, and health behaviors included in the fully adjusted model. These variables were selected because of their known strong associations with both anticholinergic and neurocognitively active medication use and CAP risk. Although all covariates were measured using routinely collected VA electronic health record data and have been used in previous studies, some degree of residual confounding due to imperfect measurement cannot be excluded. Fourth, patients in the VA tend to be older, have a higher prevalence of chronic health conditions and risk behaviors compared to the general adult US population, and are predominantly male, potentially limiting the generalizability of our findings, though our findings are consistent with previous literature including patients outside of the VA.

## 5 Conclusion

In conclusion, we found similar patterns of associations with incident CAP for both anticholinergic and non‐anticholinergic neurocognitively active medications, with higher odds observed among patients receiving multiple anticholinergic or other neurocognitively active medications. Associations were even stronger among those who reported concurrent alcohol use, particularly at‐risk and hazardous/binge levels of consumption. Together, our findings suggest the need for increased caution when prescribing these medications, especially for those who report alcohol use.

### 5.1 Plain Language Summary

Some medicines used to treat mental health and neurological conditions can affect thinking, alertness, or coordination. These include medications like antidepressants, antipsychotics, and sedatives. Some of these also have anticholinergic properties, which have been linked to a higher risk of pneumonia. Alcohol is another known risk factor for pneumonia. In this large study of over 900 000 US Veterans, we looked at whether medicines that affect the brain—with or without anticholinergic properties—were associated with hospital admission with pneumonia. We also looked at whether alcohol use influenced this risk. We found that both anticholinergic and non‐anticholinergic neurocognitive medicines were associated with higher chances of being hospitalized with pneumonia. The odds were even greater in people who reported drinking alcohol, especially at risky or hazardous levels, and those taking multiple medications at the same time. These findings suggest that alcohol and commonly prescribed neurocognitive medications may interact to raise pneumonia risk. Healthcare providers should take alcohol use into account when prescribing these medicines and consider routine screening. These results also support expanding pneumonia prevention strategies to include monitoring of and intervention on alcohol use.

## Author Contributions

A.C.J. and C.T.R. conceived the study. C.T.R. curated the data. W.H.W. and C.T.R. performed the formal analysis. A.C.J. acquired funding. J.T. and C.T.R. designed the methodology. A.C.J. and C.T.R. managed and coordinated the project. A.C.J. and C.T.R. procured resources to carry out the study. W.H.W. and C.T.R. developed programming. A.C.J. and C.T.R. provided oversight and leadership of the project. W.H.W. and C.T.R. prepared data visualizations. W.H.W. and C.T.R. wrote the first draft of the manuscript. All the authors wrote (reviewed and edited) the manuscript. The senior author attests that all listed authors meet authorship criteria and that no others meeting the criteria have been omitted.

## Funding

This work was supported by the National Institute on Alcohol Abuse and Alcoholism [P01‐AA029545, U01‐AA026224, U24‐AA020794, U01‐AA020790, U10‐AA013566]. The funders had no role in considering the study design or in the collection, analysis, interpretation of data, writing of the report, or decision to submit the article for publication.

## Conflicts of Interest

The authors declare no conflicts of interest.

## Supporting information


**Table S1:** List of anticholinergic and neurocognitively active medications included in this study.
**Table S2:** Fully parameterized model for independent associations and interactions between anticholinergic medication receipt and alcohol consumption.
**Table S3:** Fully parameterized model for independent associations and interactions between non‐anticholinergic neurocognitively active medication receipt and alcohol consumption.

## Data Availability

Due to US Department of Veterans Affairs (VA) regulations and our ethics agreements, the analytic data sets used for this study are not permitted to leave the VA firewall without a data use agreement. This limitation is consistent with other studies based on VA data. However, VA data are made freely available to researchers with an approved VA study protocol. For more information, please visit https://www.virec.research.va.gov or contact the VA Information Resource Center at virec@va.gov.
