# Supplementary material for: Concurrent Alcohol Use and the Relative Risk of Community‐Acquired Pneumonia Associated With Anticholinergic and Non‐Anticholinergic Neurocognitively Active Medication Receipt: A National Nested Case–Control Study Among US Veterans
Source: Pharmacoepidemiol Drug Saf. 2025 Dec 3;34(12):e70279. doi: 10.1002/pds.70279 (PMC12676203; doi:10.1002/pds.70279)
Supplement: Supplementary file 1 — Table S1: List of anticholinergic and neurocognitively active medications included in this study. Table S2: Fully parameterized model for independent associations and interactions between anticholinergic medication receipt and alcohol consumption. Table S3: Fully parameterized model for independent associations and interactions between non‐anticholinergic neurocognitively active medication receipt and alcohol consumption. [file PDS-34-e70279-s001.docx]

**Supplementary Appendix**

**Concurrent alcohol use and the relative risk of community-acquired pneumonia associated with anticholinergic and non-anticholinergic neurocognitively-active medication receipt: a national nested case-control study among US Veterans**

William H. Wang, Kristina Crothers, Kathleen M. Akgün, Kirsha S. Gordon, Maria C. Rodriguez-Barradas, Julie A. Womack, Jennifer Thompson, Amy C. Justice, Christopher T. Rentsch

**Table S1.** List of anticholinergic and neurocognitively-active medications included in this study

**Table S2.** Fully parameterized model for independent associations and interactions between anticholinergic medication receipt and alcohol consumption

**Table S3.** Fully parameterized model for independent associations and interactions between non-anticholinergic neurocognitively-active medication receipt and alcohol consumption

| **Table S1.** List of anticholinergic and neurocognitively-active medications included in this study | | |
| --- | --- | --- |
| **Type** | **Class** | **Medication** |
| Anticholinergic |  | ALPRAZOLAM* |
|  |  | AMITRIPTYLINE* |
|  |  | AMOXAPINE* |
|  |  | ATROPINE |
|  |  | BELLADONNA* |
|  |  | BENZATROPINE |
|  |  | BROMPHENIRAMINE |
|  |  | CARBINOXAMINE |
|  |  | CARISOPRODOL* |
|  |  | CHLORPHENIRAMINE |
|  |  | CHLORPROMAZINE* |
|  |  | CLEMASTINE |
|  |  | CLOMIPRAMINE* |
|  |  | CLORAZEPATE* |
|  |  | CLOZAPINE* |
|  |  | COLCHICINE |
|  |  | CYPROHEPTADINE |
|  |  | DARIFENACIN |
|  |  | DESIPRAMINE* |
|  |  | DICYCLOMINE |
|  |  | DIGOXIN |
|  |  | DIMENHYDRINATE |
|  |  | DIPHENHYDRAMINE |
|  |  | DOXEPIN* |
|  |  | DOXYLAMINE |
|  |  | FESOTERODINE |
|  |  | FLAVOXATE |
|  |  | FLUPHENAZINE* |
|  |  | FUROSEMIDE |
|  |  | HOMATROPINE |
|  |  | HYDROXYZINE |
|  |  | HYOSCYAMINE |
|  |  | IMIPRAMINE* |
|  |  | IPRATROPIUM |
|  |  | MAPROTILINE* |
|  |  | MECLIZINE |
|  |  | NORTRIPTYLINE* |
|  |  | OLANZAPINE* |
|  |  | ORPHENADRINE* |
|  |  | OXYBUTYNIN |
|  |  | PAROXETINE* |
|  |  | PERPHENAZINE* |
|  |  | PROCYCLIDINE |
|  |  | PROMETHAZINE* |
|  |  | PROPANTHELINE |
|  |  | PROTRIPTYLINE* |
|  |  | QUETIAPINE* |
|  |  | METOCLOPRAMIDE |
|  |  | SCOPOLAMINE* |
|  |  | SOLIFENACIN |
|  |  | THIORIDAZINE* |
|  |  | THIOTHIXENE* |
|  |  | TIZANIDINE* |
|  |  | TOLTERODINE |
|  |  | TRIFLUOPERAZINE* |
|  |  | TRIHEXYPHENIDYL |
|  |  | TROSPIUM |
| Neurocognitively-active | Opioids | OPIUM |
|  |  | BUPRENORPHINE |
|  |  | BUTORPHANOL |
|  |  | CODEINE |
|  |  | FENTANYL |
|  |  | HYDROCODONE |
|  |  | HYDROMORPHONE |
|  |  | METHADONE |
|  |  | MORPHINE |
|  |  | NALBUPHINE |
|  |  | OXYCODONE |
|  |  | OXYMORPHONE |
|  |  | TAPENTADOL |
|  |  | TRAMADOL |
|  |  | PENTAZOCINE |
|  |  | ALFENTANIL |
|  |  | LEVORPHANOL |
|  |  | MEPERIDINE |
|  |  | SUFENTANIL |
|  |  | PROPOXYPHENE |
|  | Antidepressants | CITALOPRAM |
|  |  | ESCITALOPRAM |
|  |  | FLUOXETINE |
|  |  | FLUVOXAMINE |
|  |  | PAROXETINE |
|  |  | SERTRALINE |
|  |  | VILAZODONE |
|  |  | DESVENLAFAXINE |
|  |  | DULOXETINE |
|  |  | LEVOMILNACIPRAN |
|  |  | MILNACIPRAN |
|  |  | VENLAFAXINE |
|  |  | AMITRIPTYLINE |
|  |  | AMOXAPINE |
|  |  | CLOMIPRAMINE |
|  |  | DESIPRAMINE |
|  |  | DOXEPIN |
|  |  | IMIPRAMINE |
|  |  | NORTRIPTYLINE |
|  |  | PROTRIPTYLINE |
|  |  | TRIMIPRAMINE |
|  |  | MAPROTILINE |
|  |  | ISOCARBOXAZID |
|  |  | PHENELZINE SULFATE |
|  |  | SELEGILINE |
|  |  | TRANYLCYPROMINE |
|  |  | BUPROPION |
|  |  | ESKETAMINE |
|  |  | MIRTAZAPINE |
|  |  | NEFAZODONE |
|  |  | TRAZODONE |
|  |  | VORTIOXETINE |
|  | Sedative-hypnotics | AMOBARBITAL |
|  |  | BUTABARBITAL |
|  |  | PHENOBARBITAL |
|  |  | SECOBARBITAL |
|  |  | BUTALBITAL |
|  |  | ALPRAZOLAM |
|  |  | ESTAZOLAM |
|  |  | LORAZEPAM |
|  |  | MIDAZOLAM |
|  |  | OXAZEPAM |
|  |  | TEMAZEPAM |
|  |  | TRIAZOLAM |
|  |  | CHLORDIAZEPOXIDE |
|  |  | CLONAZEPAM |
|  |  | CLORAZEPATE |
|  |  | DIAZEPAM |
|  |  | FLURAZEPAM |
|  |  | ESZOPICLONE |
|  |  | ZOPICLONE |
|  |  | ZALEPLON |
|  |  | ZOLPIDEM |
|  |  | BUSPIRONE |
|  |  | MEPROBAMATE |
|  |  | SUVOREXANT |
|  |  | DEXMEDETOMIDINE |
|  |  | LEMBOREXANT |
|  | Muscle relaxants | BACLOFEN |
|  |  | CARISOPRODOL |
|  |  | CHLORZOXAZONE |
|  |  | CYCLOBENZAPRINE |
|  |  | DANTROLENE |
|  |  | METAXALONE |
|  |  | METHOCARBAMOL |
|  |  | ORPHENADRINE |
|  |  | TIZANIDINE |
|  | Lithium | LITHIUM |
|  | Antipsychotics | CHLORPROMAZINE |
|  |  | FLUPHENAZINE |
|  |  | PERPHENAZINE |
|  |  | THIORIDAZINE |
|  |  | THIOTHIXENE |
|  |  | TRIFLUOPERAZINE |
|  |  | ARIPIPRAZOLE |
|  |  | ASENAPINE |
|  |  | BREXPIPRAZOLE |
|  |  | CARIPRAZINE |
|  |  | CLOZAPINE |
|  |  | HALOPERIDOL |
|  |  | ILOPERIDONE |
|  |  | LOXAPINE |
|  |  | LURASIDONE |
|  |  | OLANZAPINE |
|  |  | PALIPERIDONE |
|  |  | PIMAVANSERIN |
|  |  | QUETIAPINE |
|  |  | RISPERIDONE |
|  |  | ZIPRASIDONE |
|  |  | LUMATEPERONE |
|  |  | MOLINDONE |
|  | Anticonvulsants | BRIVARACETAM |
|  |  | CARBAMAZEPINE |
|  |  | CENOBAMATE |
|  |  | CLOBAZAM |
|  |  | DIVALPROEX |
|  |  | ESLICARBAZEPINE |
|  |  | ETHOSUXIMIDE |
|  |  | FELBAMATE |
|  |  | FOSPHENYTOIN |
|  |  | GABAPENTIN |
|  |  | LACOSAMIDE |
|  |  | LAMOTRIGINE |
|  |  | LEVETIRACETAM |
|  |  | METHSUXIMIDE |
|  |  | OXCARBAZEPINE |
|  |  | PERAMPANEL |
|  |  | PHENYTOIN |
|  |  | PRIMIDONE |
|  |  | RUFINAMIDE |
|  |  | TIAGABINE |
|  |  | TOPIRAMATE |
|  |  | VALPROATE SODIUM |
|  |  | VALPROIC ACID |
|  |  | VIGABATRIN |
|  |  | ZONISAMIDE |
|  |  | PREGABALIN |
|  |  | CANNABIDIOL |
|  | Anti-Parkinson's agents | AMANTADINE |
|  |  | APOMORPHINE |
|  |  | ENTACAPONE |
|  |  | ISTRADEFYLLINE |
|  |  | LEVODOPA |
|  |  | OPICAPONE |
|  |  | PRAMIPEXOLE |
|  |  | RASAGILINE |
|  |  | ROPINIROLE |
|  |  | ROTIGOTINE |
|  |  | SAFINAMIDE |
|  |  | SELEGILINE |
|  |  | TOLCAPONE |
|  | Antivertigo agents | SCOPOLAMINE |
|  | Antihistamines | PROMETHAZINE |
|  |  | CARBINOXAMINE |
|  |  | CLEMASTINE |
|  |  | DIMENHYDRINATE |
|  |  | DIPHENHYDRAMINE |
|  |  | CHLORPHENIRAMINE |
|  |  | HYDROXYZINE |
|  |  | CYPROHEPTADINE |
|  |  | DOXYLAMINE |
|  |  | BROMPHENIRAMINE |
|  |  | DEXCHLORPHENIRAMINE |
|  |  | MECLIZINE |
|  |  | LEVOCETIRIZINE |
|  |  | CETIRIZINE |
|  |  | DESLORATADINE |
|  |  | FEXOFENADINE |
|  |  | LORATADINE |
|  | Amphetamine-derivates | DEXMETHYLPHENIDATE |
|  |  | METHYLPHENIDATE |
|  |  | DEXTROAMPHETAMINE |
|  |  | AMPHETAMINE |
|  |  | AMPHETAMINE RESIN COMPLEX |
|  |  | LISDEXAMFETAMINE |
|  |  | METHAMPHETAMINE |
|  | Other | BELLADONNA |
|  |  | ATOMOXETINE |
|  |  | DEXTROMETHORPHAN |
|  |  | METOCLOPRAMIDE |
|  |  | BENZTROPINE |
|  |  | DICYCLOMINE |
|  |  | HYOSCYAMINE |
|  |  | PROCYCLIDINE |
|  |  | PROPANTHELINE |
|  |  | TRIHEXYPHENIDYL |
| *=classified as anticholinergic and neurocognitively-active | | |

| **Table S2.** Fully parameterized model for independent associations and interactions between anticholinergic medication receipt and alcohol consumption | | |
| --- | --- | --- |
|  | **Minimally adjusted OR (95% CI)** | **Fully adjusted OR (95% CI)** |
| **Independent Associations** |  |  |
| Anticholinergic medication receipt |  |  |
| No | 1.00 (ref) | 1.00 (ref) |
| Yes | 3.13 (3.04-3.21) | 1.62 (1.57-1.67) |
| Alcohol consumption |  |  |
| Abstinent | 1.97 (1.94-2.00) | 1.71 (1.67-1.74) |
| Low-risk | 1.00 (ref) | 1.00 (ref) |
| At-risk | 1.07 (1.04-1.11) | 1.03 (1.00-1.06) |
| Hazardous/binge | 2.54 (2.42-2.66) | 1.77 (1.68-1.86) |
| Missing | 0.60 (0.59-0.62) | 0.61 (0.59-0.62) |
| **Interactions** |  |  |
| Anticholinergic medication receipt 𝑥 alcohol consumption | |  |
| Yes 𝑥 abstinent | 0.79 (0.77-0.82) | 0.81 (0.78-0.84) |
| Yes 𝑥 at-risk | 1.05 (0.99-1.10) | 1.04 (0.98-1.11) |
| Yes 𝑥 hazardous/binge | 0.75 (0.69-0.82) | 0.74 (0.67-0.82) |
| Yes 𝑥 missing | 1.06 (1.00-1.11) | 1.10 (1.04-1.16) |
| **Covariates** |  |  |
| Smoking status |  |  |
| Never |  | 1.00 (ref) |
| Former |  | 1.07 (1.05-1.09) |
| Current |  | 1.84 (1.81-1.88) |
| Alcohol use disorder |  | 1.46 (1.42-1.49) |
| Asthma |  | 1.00 (0.97-1.03) |
| Cardiovascular disease |  | 1.17 (1.15-1.19) |
| Chronic obstructive pulmonary disease |  | 2.06 (2.03-2.10) |
| Diabetes |  | 0.72 (0.71-0.73) |
| Chronic hepatitis C virus infection |  | 1.26 (1.22-1.30) |
| Charlson Comorbidity Index score |  |  |
| 0 |  | 1.00 (ref) |
| 1-2 |  | 1.74 (1.71-1.78) |
| 3-4 |  | 2.66 (2.60-2.73) |
| ≥5 |  | 4.61 (4.48-4.74) |
| Corticosteroid receipt in previous year |  | 1.34 (1.32-1.36) |
| Influenza vaccination receipt in previous year |  | 0.97 (0.96-0.98) |
| Pneumococcal vaccination receipt in previous 5 years |  | 1.05 (1.03-1.07) |
| Number of other chronic medications* |  |  |
| 0 |  | 1.00 (ref) |
| 1-5 |  | 0.73 (0.71-0.75) |
| 6-10 |  | 1.20 (1.18-1.23) |
| ≥11 |  | 2.31 (2.25-2.36) |

| **Table S3.** Fully parameterized model for independent associations and interactions between non-anticholinergic neurocognitively-active medication receipt and alcohol consumption | | |
| --- | --- | --- |
|  | **Minimally adjusted OR (95% CI)** | **Fully adjusted OR (95% CI)** |
| **Independent Associations** |  |  |
| Non-anticholinergic neurocognitively-active medication receipt |  |  |
| No | 1.00 (ref) | 1.00 (ref) |
| Yes | 2.74 (2.68-2.81) | 1.61 (1.57-1.66) |
| Alcohol consumption |  |  |
| Abstinent | 2.00 (1.96-2.04) | 1.76 (1.72-1.79) |
| Low-risk | 1.00 (ref) | 1.00 (ref) |
| At-risk | 1.05 (1.02-1.09) | 1.02 (0.98-1.06) |
| Hazardous/binge | 2.83 (2.67-3.00) | 1.98 (1.86-2.11) |
| Missing | 0.63 (0.61-0.64) | 0.60 (0.58-0.62) |
| **Interactions** |  |  |
| Non-anticholinergic neurocognitively-active medication receipt 𝑥 alcohol consumption | |  |
| Yes 𝑥 abstinent | 0.85 (0.82-0.87) | 0.83 (0.80-0.86) |
| Yes 𝑥 at-risk | 1.07 (1.02-1.13) | 1.06 (1.01-1.12) |
| Yes 𝑥 hazardous/binge | 0.70 (0.65-0.76) | 0.69 (0.63-0.75) |
| Yes 𝑥 missing | 1.07 (1.02-1.12) | 1.14 (1.09-1.20) |
| **Covariates** |  |  |
| Smoking status |  |  |
| Never |  | 1.00 (ref) |
| Former |  | 1.07 (1.05-1.09) |
| Current |  | 1.83 (1.80-1.86) |
| Alcohol use disorder |  | 1.44 (1.41-1.48) |
| Asthma |  | 1.00 (0.97-1.03) |
| Cardiovascular disease |  | 1.21 (1.19-1.23) |
| Chronic obstructive pulmonary disease |  | 2.09 (2.05-2.12) |
| Diabetes |  | 0.72 (0.71-0.74) |
| Chronic hepatitis C virus infection |  | 1.26 (1.22-1.30) |
| Charlson Comorbidity Index score |  |  |
| 0 |  | 1.00 (ref) |
| 1-2 |  | 1.74 (1.70-1.77) |
| 3-4 |  | 2.66 (2.60-2.73) |
| ≥5 |  | 4.61 (4.48-4.74) |
| Corticosteroid receipt in previous year |  | 1.32 (1.30-1.34) |
| Influenza vaccination receipt in previous year |  | 0.96 (0.95-0.98) |
| Pneumococcal vaccination receipt in previous 5 years |  | 1.05 (1.03-1.07) |
| Number of other chronic medications* |  |  |
| 0 |  | 1.00 (ref) |
| 1-5 |  | 0.68 (0.66-0.70) |
| 6-10 |  | 1.11 (1.08-1.14) |
| ≥11 |  | 2.13 (2.07-2.18) |
